# How to implement Illness Management and Recovery (IMR) in mental health service settings: evaluation of the implementation strategy

**DOI:** 10.1186/s13033-017-0120-z

**Published:** 2017-01-23

**Authors:** Karina Myhren Egeland, Torleif Ruud, Terje Ogden, Rickard Färdig, Jonas Christoffer Lindstrøm, Kristin Sverdvik Heiervang

**Affiliations:** 1 Division of Mental Health Services, Akershus University Hospital, Sykehusveien 25, 1478 Lørenskog, Norway; 2 Institute of Clinical Medicine, University of Oslo, Oslo, Norway; 3 Norwegian Center for Child Behavioral Development, Essendropsgate 3, 0368 Oslo, Norway; 4 Institute of Psychology, University of Oslo, Oslo, Norway; 5 Department of Neuroscience, Psychiatry, Uppsala University, Uppsala, Sweden; 6 Helse Sør-Øst Health Services Research Centre, Akershus University Hospital, Sykehusveien 25, 1478 Lørenskog, Norway

**Keywords:** Fidelity, Implementation strategies, Feasibility, Illness Management and Recovery

## Abstract

**Background:**

The purpose of this study was to evaluate the implementation strategy used in the first-phase of implementation of the Illness Management and Recovery (IMR) programme, an intervention for adults with severe mental illnesses, in nine mental health service settings in Norway.

**Methods:**

A total of 9 clinical leaders, 31 clinicians, and 44 consumers at 9 service settings participated in the implementation of IMR. Implementation was conducted by an external team of researchers and an experienced trainer. Data were gathered on fidelity to the intervention and implementation strategy, feasibility, and consumer outcomes.

**Results:**

Although the majority of clinicians scored within the acceptable range of high intervention fidelity, their participation in the implementation strategy appeared to moderate anticipated future use of IMR. No service settings reached high intervention fidelity scores for organizational quality improvement after 12 months of implementation. IMR implementation seemed feasible, albeit with some challenges. Consumer outcomes indicated significant improvements in illness self-management, severity of problems, functioning, and hope. There were nonsignificant positive changes in symptoms and quality of life.

**Conclusions:**

The implementation strategy appeared adequate to build clinician competence over time, enabling clinicians to provide treatment that increased functioning and hope for consumers. Additional efficient strategies should be incorporated to facilitate organizational change and thus secure the sustainability of the implemented practice.

*Trial registration* ClinicalTrials.gov NCT02077829. Registered 25 February 2014

## Background

Although there is a continued growth in knowledge on how to successfully implement innovations in health care, research has been hampered by the varied quality of reports on implementation process [1]. Strategies are not described in detail or justified, thus it remains challenging to bring evidence-based practices to service users who would benefit from them [2, 3].

Implementation outcomes (e.g. fidelity) result from deliberate and purposeful actions to implement new interventions, and serve as indicators of the level of implementation success [4]. Much research has been performed on the fidelity of evidence-based interventions (i.e., the degree to which the interventions were implemented as intended in the original programme) [4]. However, the fidelity of implementation strategies (i.e., methods or techniques, single or multifaceted, used to enhance the implementation of the innovation) remains underreported in the health literature [2, 3]. In addition to implementation outcomes, consumer outcomes are the most important criteria for evaluating both intervention and implementation strategies [4]. If we fail to improve consumer well being, we need to reconsider our intervention or implementation strategy.

Illness Management and Recovery (IMR) is a standardized psychosocial intervention with a strong empirical foundation in illness self-management and recovery, and is based on the stress-vulnerability model [5, 6]. It was developed during the National Implementing Evidence-Based Practices (NIEBP) project in the USA [7] and is designed to help people with serious mental illnesses manage their illness and achieve personal goals [8]. Five strategies form the basis of the IMR programme: psychoeducation to improve knowledge of mental illness, relapse prevention to reduce relapses and rehospitalisation, behavioural training to improve medication adherence, coping skills training to reduce the severity and distress of persistent symptoms, and social training to strengthen social support. Clinicians teach these strategies through a combination of educational, motivational, and cognitive-behavioural techniques [5, 9]. IMR is organized into 11 modules with different topics. A workbook with educational handouts has been developed and is taught weekly to service users individually or in groups, for 10–12 months. A review [8] showed IMR is advantageous to treatment as usual, according to observer ratings of psychiatric symptoms, as well as consumer and clinician ratings. Two randomized studies with active control groups have found significant improvements but no significant differences between the groups [10, 11]. However, the studies had weaknesses such as low participation rates, non-blinded staff and high drop out rates.

Based on experiences from the NIEBP project, a toolkit was developed to guide the implementation of several evidence-based practices, including IMR [12, 13]. This includes strategies such as informational and training materials, implementation recommendations, and measurements to facilitate use of the programme. The toolkit has not been statistically tested and evaluated. Studies examining the implementation of IMR have generally used these strategies, which include IMR-specific training and supervision, intervention fidelity monitoring [8, 10, 11], as well as external facilitation such as in situ audits [14] or technical assistance [7, 15, 16]. An essential weakness of these studies is the lack of documentation and reporting on fidelity to the implementation strategies. Moreover, the strategies resulted in mixed implementation outcomes. Although higher fidelity to interventions has been associated with better consumer outcomes [17], the level of fidelity has varied widely in several multisite studies [15, 17], and showed reduced sustainability over time [7, 18]. The need for organizational-level changes, including programme leadership, has also been reported [15, 19, 20]. IMR seems to be feasible (i.e., the extent to which a practice can be used or carried out within a setting, often based on consumer retention and participation) but challenging to implement. The curriculum is comprehensive and completion rates vary substantially between studies (18–30%) [10, 21]. Dropout rates (*Mdn* = 24%) and completion rates (*Mdn* = 63%) could be improved [8]. Details about implementation strategy fidelity were lacking in earlier studies, making it difficult to draw conclusions about whether the implementation outcomes were a result of the intervention or the implementation strategies.

This study evaluates a multi-faceted strategy used to implement IMR in nine Norwegian mental health service settings and covers the first 18 months of implementation. Proctor et al.’s [3] recommendations on specifying and reporting strategies were used to operationalize the implementation strategy. Seven dimensions were used to define adequate operationalization of the implementation strategies (see Table 1). Data were gathered on fidelity to intervention, implementation strategy, feasibility, and consumer outcomes.

**Table 1:** Description of the multi-faceted implementation strategy

Intervention	Actor	Action	Action target	Temporality^a^	Dose	Implementation outcome targeted^b^	Justification
Introductory seminar	Intervention developer	The IMR programme was introduced with introductory video and PowerPoint presentation	Motivate clinicians and organizations to prepare for implementation in organizations	Preparation	One-day seminar	Adoption	Rogers [22] Knowledge as the first step to change
Initiate leadership	External implementation team	The external team had individual meetings with leaders to discuss the implementation process and the research project	Service leaders initiate change in organization to facilitate quality improvement	Preparation	One meeting per service	Feasibility fidelity	Innovative, supportive leaders as important for successful implementation [23]
Coordinator recruitment	Service leader	Leaders were asked to choose a coordinator among staff to advocate for the programme	To have coordinators advocate for or champion the implementation of IMR	Preparation		Feasibility fidelity	Champions as a driving force behind implementation [24]
Distribute educational materials	External implementation team	Distribution of the IMR manual [6] to support clinical care	To increase clinicians’ knowledge and skills of intervention	Implementation		Fidelity	Educational materials better than no materials [25]
Ongoing training	IMR trainer	To teach clinicians about the IMR in an ongoing way	To increase clinicians’ knowledge and skills of intervention	Implementation	Four days of training + two booster sessions	Fidelity	Ongoing training better than single one-time strategies [26, 27]
Clinical consultations	IMR trainer	Answer questions, review case implementation, make suggestions, and provide encouragement	To increase clinicians’ knowledge and skills to use the innovation	Implementation	20 min per week in group by phone for 9 months, then biweekly for 5 months	Fidelity	Post-training consultations more important than quality of/type of training [28]
Audit and feedback in consultations	IMR trainer	IMR trainer rated audiotaped sessions and gave verbal and written feedback	Clinicians’ understanding and ability to break down the intervention into more doable steps	Implementation	First session in every module audiotaped and rated	Fidelity Feasibility	a&f leads to improvements in professional practice [29]
Process monitoring and feedback	External implementation team	Implementation process was assessed after 6 and 12 months and verbal and written feedback was given	To improve the quality of the programme delivery, to prevent drift and maximize effectiveness	Implementation	After six and 12 months of implementation	Fidelity Feasibility	Monitoring can prevent drift and maximize effectiveness [30]
Outcome monitoring	Clinicians	Consumer outcomes (IMRS) were assessed at the end of every module. Clinicians were encouraged to evaluate the outcomes continuously	To improve the quality of the programme delivery, to prevent drift and maximize effectiveness	Implementation	After each module	Fidelity feasibility	Monitoring can prevent drift and maximize effectiveness [30]

^a^Temporality: Based on McGovern et al.’s [19] three stages; preparation, implementation, and maintenance

^b^Implementation outcome targeted: Based on outcomes presented in Proctor et al.’s [4] paper

This is the first IMR implementation study to report clinician participation in the implementation strategy as a measure of implementation fidelity, which is essential for capturing whether the strategy in question increases clinician uptake of the intervention. Also, by thoroughly reporting on the implementation strategy we are more able to draw conclusions on the implementation outcomes. This will benefit future IMR implementation and research. The research question was: Did the implementation strategy facilitate implementation of IMR in the service settings?

## Methods

### Design

The study used an observational prospective design. An implementation strategy was introduced while observations and information gathering (intervention process, outcomes) were performed. The information was also used to actively enhance implementation efforts during the course of the study. The study was approved by the regional committees for medical and health research ethics [REK 2013/2035].

### Participants

The IMR programme implementation took place between November 2013 and June 2015. Seven primary care service settings and six specialized mental healthcare services located in one of Norway’s most populated areas were invited to participate. Seven primary care service settings and two specialized service settings accepted the invitation.

All nine clinical service leaders took part in the implementation process, which included six women and three men. Of the 138 employees in the nine participating service settings (*Mdn* = 12 per service, range 9–31), 36 clinicians participated in IMR implementation. Five withdrew during the implementation period (four changed position), leaving 31 clinicians in the study (*Mdn* = 4 per service, range 2–5). The clinicians were mostly female (*n* = 21), and the mean age was 44 years (*SD* = 9.1). The mean years of clinical experience was 11.8 (*SD* = 8.3). Clinician disciplines included nursing/social education (*n* = 15), social work (*n* = 8), physiotherapy/pedagogy (*n* = 7), and psychology (*n* = 1). Most had a bachelor’s degree (*n* = 27) and the remainder had a master’s degree (*n* = 4).

Consumers were recruited by clinicians using the IMR programme’s intake criteria (i.e., symptoms of or diagnosed with severe mental illness). The services considered that a large portion (10–100%) of their consumers were eligible of receiving IMR. Clinicians were asked to recruit at least 1 consumer each. There were 44 consumers who signed the informed consent to participate in the research. Twenty-eight were males, and the mean age was 40.7 (*SD* = 10.4). Their main diagnoses were schizophrenia (*n* = 17), bipolar disorder (*n* = 9), depression (*n* = 4), other (*n* = 5), missing (*n* = 3) or non-diagnosed (*n* = 6). Their occupations were unemployed (*n* = 27), in vocational rehabilitation (*n* = 11), employed (*n* = 3), or homemaker/sick leave (*n* = 3). Based on consumer choice or service decisions, 27 were included in the IMR groups and the remaining 17 had IMR on an individual basis.

### Implementation process

An external team of two researchers (KE and KSH) responsible for the implementation process served as an advisory group for the service settings. A psychologist (RF) with extensive experience in IMR, both as a practitioner and as a trainer, was responsible for training and supervising the clinicians.

The strategy used to implement IMR was based on the recommended implementation strategies from the IMR toolkit [12, 13] (Table 1). As justified by Rogers’ theory, which describes knowledge as the first step to change [22], a 1-day introductory seminar was held by one of the developers of the model 6 months prior to implementation to inform the service settings about the content of IMR. Enrolment in the project took place thereafter. As supportive, innovative leaders have been shown to be important for successful implementation [23], the external team held individual meetings with clinical leaders prior to the training. The implementation process and research project were discussed. Champions have been seen as a driving force behind implementation [24], and leaders were asked to identify a staff member to advocate for the programme. Champions were expected to serve as a link between clinical leadership and IMR clinicians. Two clinical leaders opted to serve as champions, as they were also attending the IMR training.

As educational materials have shown a small beneficial effect on professional practice outcomes [25], the external team distributed educational materials [6] to all participating clinicians prior to training, including information brochures to help introduce IMR to consumers. Based on training frequency recommendations [26, 27], training occurred in two 2-day seminars over 1 month, plus two booster sessions the following year. The training content shifted between lectures on core skills and strategies and exercises to practice the techniques. The booster sessions focused on solving specific challenges in using and implementing the programme.

After the initial 4 days of training, clinicians began recruiting consumers to participate in IMR. All but one was recruited within 5 months, and the last one after 8 months. Based on research supporting post-training consultations [28], clinicians began weekly telephonic group consultations with the IMR trainer. As feedback can lead to improvements in professional practice [29], clinicians were asked to audiotape the first session in every IMR module (11 modules altogether). The IMR trainer rated these sessions and provided verbal and written feedback. Weekly consultations continued for approximately 9 months, and then shifted in biweekly for another 5 months. No local adaptations to the IMR manual were allowed. The consultations concluded in June 2015.

As monitoring can prevent drift and maximize effectiveness [30], the process was monitored in every service setting after 6 and 12 months of implementation. Clinical leaders and clinicians received verbal and written feedback, with recommendations for improving implementation. In addition, the clinicians were encouraged to evaluate consumer outcomes after each IMR module.

### Measures

#### Implementation outcomes

Three measures were used to assess intervention fidelity. The Illness Management and Recovery Fidelity Scale (IMR fidelity) [19] is a 13-item scale that assesses the implementation of specific strategies within IMR programme (e.g., motivational and cognitive-behavioural techniques), and structural and curriculum-based elements (e.g., the number of sessions held or content modules covered). A summed and averaged fidelity score of 4 or more = successful implementation, 3–4 = moderate fidelity, <3 = low fidelity [7, 31]. The scale has shown high interrater reliability with other fidelity scales, and sensitivity to increased scores after training and consultation [31].

The General Organizational Index (GOI) is a 12-item scale measuring the general quality of the clinical care [32]. It consists of two subscales measuring quality improvement at the organizational level (i.e., existence of training and supervision facilities, process and outcome monitoring, and quality assurance) and at the consumer level (i.e., provision of individualized eligibility determination, assessment, treatment plan, treatment, and choice regarding service provision). In addition, penetration (the extent to which the practice is offered) and understanding of and commitment to programme philosophy are measured. The scale has shown adequate psychometric properties [32].

The IMR fidelity and GOI were translated by KE and KSH and have not been validated in a Norwegian context. They completed the IMR fidelity and GOI ratings during a daylong site visit by interviewing leaders, clinicians, and consumers after 6 and 12 months of implementation. IMR sessions were observed, chart reviews examined, and IMR educational handouts reviewed. The raters independently assessed the programme and compared ratings. Discrepancies were resolved through discussions with each other and with staff.

While the IMR fidelity focuses primarily on structural aspects of the IMR programme or clinician skills at the service level, The Illness Management and Recovery Treatment Integrity Scale (IT-IS) [33] measures clinicians’ individual competence in providing the programme, that is the quality of the programme delivery [4]. The 16-item scale has shown a one-factor model with good internal consistency [33] and excellent interrater reliability (α = .92). IT-IS was rated by the trained rater (RF), using audiotapes of the clinicians’ IMR sessions. Clinicians’ ability to deliver audiotapes was also registered.

To measure fidelity to the implementation strategy, clinicians’ participation in the implementation process was assessed through training and consultation attendance rates. The number of IMR consumers that were recruited was also recorded.

After 12 months of implementation, the clinicians were asked on a 5-point scale (0 = *not at all*, 4 = *to a very great extent*) whether they would continue using IMR.

In terms of feasibility, data was gathered on consumer retention and participation in IMR.

#### Consumer outcomes

Consumers filled out a paper questionnaire at the time of IMR implementation initiation and at the end of the implementation period. The Illness Management and Recovery scale (IMRS) [19] is a 15-item scale that assesses illness self-management. It measures consumer behaviour towards core components in the IMR programme. A higher score indicates better functioning. The scale includes parallel clinician and consumer versions, and has shown satisfactory internal reliability and strong test–retest reliability [34]. It was translated into Norwegian by KSH and KE and has not been validated in a Norwegian context.

Health of the Nation Outcome Scale (HoNOS) [35] measures consumer problem severity based on behaviour, impairment, symptoms, and social functioning. Clinicians rate consumers on a 12-item scale (0 = *no severity*, 4 = *high severity*), which is designed to measure change in response to an intervention. Internal consistency has been moderate (α = .59–.76) and it shows fair to moderate test–retest reliability [36].

The split version of the Global Assessment of Functioning (S-GAF) [37] was used by clinicians to rate consumer functioning on two 1-point scales (1 = *low functioning*, 100 = *high functioning*), one score for symptoms and one for functioning. The two scores have been found to be highly generalizable [38].

The Adult State Hope scale (ASHS) [39] is a six-item self-rated measure of hope that is scored on a 7-point scale (1 = *definitely false*, 7 = *definitely true*). It has demonstrated internal consistency, high levels of convergent and discriminant validity, and good sensitivity.

Quality of Life (QoL5) [40] is a 5-item self-rated measure of consumers’ subjective, objective, and existential quality of life, scored on a 5-point scale (1 = *very high*, 5 = *very low*). It has shown acceptable internal consistency and sensitivity.

The Client Satisfaction Questionnaire (CSQ-8) [41] measures consumer satisfaction with services on an 8-point scale (1 = *low satisfaction*, 4 = *high satisfaction*). In this study, the questions assessed satisfaction with IMR after the implementation period. The scale has shown high internal consistency [42].

### Data analyses

There were few missing items on the participants’ questionnaires altogether (18 items = .46% in total). When no more than two items were missing, values were replaced with the mean value of the scale or subscale. To compare services during and after implementation, and to compare clinician- and consumer-rated outcomes pre- and post-implementation, paired samples *t* tests with bootstrapping were performed in SPSS (version 21). To examine associations between clinician participation and their intention to further use of IMR, path analysis was performed using the lavaan R package [43]. Multiple regression analyses were performed in SPSS (version 21) to examine whether higher intervention fidelity was associated with better consumer outcomes.

## Results

### Implementation outcomes

Of the nine service settings, one had difficulty implementing IMR. The clinicians could not recruit consumers, and they dropped out of consultation after 7 months. They also had the lowest score on IMR fidelity after 6 months (*M* = 3.38). Because of missing data on the IMR fidelity and GOI, this service was excluded from these two analyses. The eight remaining service settings reached a high score on the IMR fidelity scale after 6 months of implementation (*M* = 4.09, *SD* = .16, range 3.85–4.31). After 12 months all service settings had significantly improved their scores (*M* = 4.61, *SD* = .18; *M*_*diff*_ = .52, 95% BCa CI [.413, .625], *p* = .001, range 4.23–4.77).

After 6 months of implementation, the eight service settings’ mean GOI score was 2.70 (*SD* = .22, range 2.50–3.08). After 12 months all service settings had significantly improved their scores (*M* = 2.99, *SD* = .22; *M*_*diff*_ = .29, 95% BCa CI [.198, .375], *p* = .006, range 2.83–3.50).

Of the 31 participating clinicians, 20 obtained consent to audiotape sessions with consumers, and therefore had IT-IS scores. Sixty recordings were scored and the mean value was 3.54 (*SD* = .68, range 2.0–4.62), which corresponded to a score between *satisfactory* and *very good*. Comparing clinicians’ scoring on their first (*M* = 3.26, *SD* = .64) and last recording (*M* = 3.91, *SD* = .46) showed a significant improvement over time (*M* = .65, 95% BCa CI [.366, .906], *p* = .001).

In terms of fidelity to the implementation strategy, clinicians’ participation in the strategy varied extensively. Mean participation in ongoing training was 4.7 days (range 1–6, *SD* = 1.57) and 18.37 sessions for consultations (range 4–32, *SD* = 8.82). Two clinicians did not recruit any consumers and seven did not obtain consumer concent to participate in the research. On average, clinicians recruited 1.4 consumers each (range 0–5, *SD* = 1.3). After the implementation period the majority of clinicians reported they would continue to use IMR to a *great* or *very great extent* (61.2%). One-third would continue to use it to a *moderate extent* (32.3%), while two would not continue its use or use it to a *small* extent (6.5%).

**Fig. 1:**
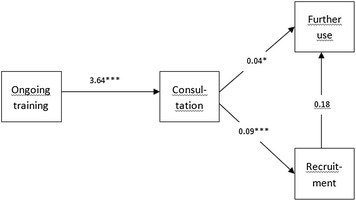
Path analysis of associations between clinician participation and the intention further use of the IMR. Ongoing training = clinicians’ participation in ongoing training. Consultation = clinicians’ participation in consultations. Recruitment = Clinicians’ consumer recruitment. Further use = Clinicians; further use of the IMR. *p < .5 , **p < .01, ***p < .001

A path analysis showed an association between clinician participation in ongoing training and consultations, which again was associated with intentions to further IMR use (Fig. 1). The more clinicians participated in training and consultation, the more likely they were to report intent to continue IMR use after the implementation period. Participation in consultations was also associated with the number of consumers recruited, but this was not associated with intentions to continue IMR use. The model fit indices were acceptable (CFI = .975; TLI = .926; RMSEA = .126; SRMR = .045).

In terms of feasibility, nine of the 44 consumers dropped out during implementation (20.5%), of which six did not start IMR. Dropouts were younger (*M* = 33, *SD* = 7.1) than the completers (*M* = 42.5, *SD* = 10.3), mostly unemployed (*n* = 7), and had no identified diagnosis (*n* = 5). The majority had problems with drug use (*n* = 5 of 9), compared to the minority of the fulfillers (*n* = 4 of 35). By the end of the implementation period, due to the varying starting times of IMR at the service settings, the consumers had received IMR for various lengths of time (*Mdn* = 14 months, range 8–16). Their participation also varied (*Mdn* = 30.5 sessions, range 7–56), as did completion rates (*Mdn* = 7 modules finished, range 2–11).

### Consumer outcomes

On clinician-rated questionnaires consumers showed significant improvements in illness management skills, problem severity as measured by behaviour, impairment, symptoms, as well as social and consumer functioning (Table 2). There was no significant decrease in mental health symptoms as measured by GAF-S. The dropouts did not significantly differ on any of the variables at baseline.

**Table 2:** Clinician- and consumer-rated outcomes pre and post implementation period

n	Variable	Time 1*		Time 2*		*M*	*p*	95% CI	
		*M*	*SD*	*M*	*SD*			*LL*	*UL*
	*Clinician rating*								
34	Illness management skills (IMRS clinician)	3.21	.348	3.58	.414	.36	.001	.202	.521
34	Severity of problems (HoNOS)	.972	.418	.736	.304	−.23	.015	−4.13	−.56
35	Consumer functioning (GAF-F)	51.06	9.36	56.66	12.6	5.6	.012	1.48	9.84
35	Consumer symptom (GAF-S)	54.97	8.34	55.43	11.8	.46	.853	−3.88	5.38
	*Consumer rating*								
35	Illness management skills (IMRS consumer)	3.07	.350	3.58	.426	.512	.001	.359	.669
35	Hope (ASHS)	3.62	1.39	4.79	.856	1.2	.001	.779	1.54
35	Quality of life (QoL5)	3.25	.605	3.14	.512	−.11	.065	−.229	.010
34	Satisfaction with services (CSQ-8)	–	–	3.24	.471	–	–	–	–

*CI* confidence interval

* Time 1 = at the time of IMR start-up. Time 2 = at the end of implementation period

On self-rated questionnaires, consumers showed significant improvements in illness management skills and hope (Table 2). There was a nonsignificant increase in the QoL5. Consumers were highly satisfied with the programme.

Looking at IMRS clinician and consumer at the end of the implementation period, increased intervention fidelity had a positive effect when adjusted for IMRS at start. Estimated increase were 2.97 IMRS points (clinician score) and 6.26 IMRS points (consumer score) per point increase in intervention fidelity. However, the results were nonsignificant (Table 3).

**Table 3:** Regression of post-implementation IMRS controlling for IMR fidelity after 12 months and baseline IMRS

Variable	IMRS clinician Time 2* (*R* ^*2*^ = .021)			IMRS consumer Time 2* (*R* ^*2*^ = .07)		
	β	*SE*	*p*	β	*SE*	*P*
IMR fidelity	.084	6.14	.632	.171	6.34	.331
IMRS Time 1*	.281	.207	.115	.236	.214	.183

Time 1 = at the time of IMR start-up. Time 2 = at the end of implementation period (two-tailed)

* p < .05. ** p < .01. *** p < .001

## Discussion

This study examined whether the chosen implementation strategy facilitated IMR implementation in Norwegian mental health service settings. Based on clinicians’ intervention fidelity to IMR, as measured by the IMR fidelity and IT-IS, the results suggest the implementation strategy was adequate for achieving high intervention fidelity among clinicians. The IMR fidelity reached scores defined as *successful implementation* [7] in eight of nine service settings, specific therapeutic techniques and structural curriculum-based elements of IMR were in place after 6 months and continued to improve during the next 6 months. IMR fidelity scores did not vary widely among the service settings as it had in earlier studies [15, 17], presumably because a contemporaneous implementation strategy was conducted by the same external implementation team and trainer. Individual clinician competence in providing IMR was also satisfactory and improved over time. However, only 20 of the 31 clinicians were evaluated on the IT-IS scale; competence is unknown for two clinicians who recruited zero consumers and nine clinicians who did not obtain consumer consent to audiotape.

The inability to audiotape may indicate low fidelity to the implementation strategy component involving audit and feedback. Wide variation occurred in clinician participation in training and consultation, as well as consumer recruitments. However, clinicians who were more engaged in the implementation strategy were more likely to report intended future use, whereas clinicians that scored the lowest on IMR fidelity after 6 months did not recruit any consumers, participated in fewer consultations, and reported lower intended future use. It is possible that clinician participation in the implementation strategy is a moderator for future IMR use. This finding points to the importance of reaching high implementation strategy fidelity, and not just high intervention fidelity. Intended future use should count as a criterion of successful implementation. A positive finding was that most clinicians reported moderate to high intentions for future IMR use after the implementation period. Given that this intention is determined by their participation in the implementation strategy, increased awareness towards fidelity to the strategy during implementation is crucial. Perhaps improved fidelity will contribute to increased sustainability, which has been shown to be a challenge in IMR implementation [7, 18]. As this study examined intention for future use and not actual use, more research should examine this further.

Similar to other studies [15, 19], it was more challenging to generate change at the organizational level than at the clinician level. In contrast to clinicians’ intervention fidelity to IMR, the quality of organizational care as measured by the GOI was low after 6 and 12 months, despite a significant improvement between the time points. This might indicate a lack of implementation strategies for facilitating organizational change. Further efforts to implement IMR should consider increasing clinical leadership’s involvement in the process, which has shown to be an important factor affecting implementation [20, 44] and building networks within the organization to promote a shared vision for implementing IMR.

In terms of feasibility, the drop-out rate (20.5%) was lower than in earlier studies (*Mdn* = 24%) [8]. Even though most consumers received IMR for more than 1 year, they only finished a median of seven modules. This may support prior findings that suggest the curriculum is demanding [8]. However, slow progression might also be due to the fact that the clinicians and the units were inexperienced with the programme prior to implementation, which could have stalled progression. Moreover, many consumers in the target group had symptoms and difficulties that might have hindered regular attendance or necessitated extended follow-up. Nevertheless, the consumers expressed satisfaction with IMR. Accordingly, IMR seems feasible, although with some challenges. The IMR implementation would perhaps have benefited from addressing feasibility more in the implementation strategy. Future implementation strategies could identify the ways in which IMR could be tailored to local needs and clarify which elements must be maintained to preserve fidelity. To promote recruitment and avoid consumer dropouts, consumers and family members could have been more involved in the implementation effort.

Although challenges in the implementation strategy were identified, outcomes indicated improvements in consumer outcomes, as was found in earlier research [15, 21]. Consumers improved significantly in illness self-management, reduced severity of problems, functioning, and hope, and experienced positive changes in symptoms as measured by the GAF-S and QoL5. In addition, we found positive, but non-significant, associations between intervention fidelity and consumer outcomes. This nonsignificant finding could be attributed to the small sample size of the study. Although nonsignificant, the direction of the tendency is the same as reported in an earlier study [17]. These results suggest that, as long as clinicians adhere to the defining principles of the IMR programme, consumer outcomes will improve. However, weaknesses in the implementation strategy at the organizational level may hinder programme sustainability at the organizational level. This might reduce likelihood that IMR is offered to other consumers and contribute disintegration of the programme.

The current study has some limitations. It used an observational design with no control group, which limits the conclusions that can be drawn from the implementation strategy and consumer data. Several instruments have not been validated in a Norwegian context. The number of participants was too low to perform subgroup analyses. Furthermore, implementation only lasted for 18 months, which is a short time period for evaluating the sustainability of the implementation of the programme. Further research should evaluate other implementation outcomes, which can shed light on the strategies used, such as acceptability, appropriateness, and implementation cost [4].

## Conclusions

This study evaluated a multi-faceted implementation strategy used to facilitate IMR implementation in nine mental health service settings. The findings suggest that the implementation strategy was adequate for building clinician competence over time, as well as increasing consumer functioning and hope. However, as clinicians’ participation in the implementation strategy seemed to operate as a moderator for their further use, increased awareness of fidelity to the implementation strategy may be critical. The implementation effort also appeared to lack strategies to facilitate organizational change and to increase the feasibility of implementing IMR. Building on the results of this evaluation, further attempts to implement IMR could lead to more efficient implement strategies, which will in turn lead to successful implementation of IMR and other evidence-based practices.

## Abbreviations

IMR: Illness Management and Recovery
NIEBP: The National Implementing Evidence-Based Practices project
GOI: The General Organizational Index
IT-IS: The Illness Management and Recovery Treatment Integrity Scale
IMRS: Illness Management and Recovery Scale
GAF: Global Assessment of Functioning
QoL5: quality of life
